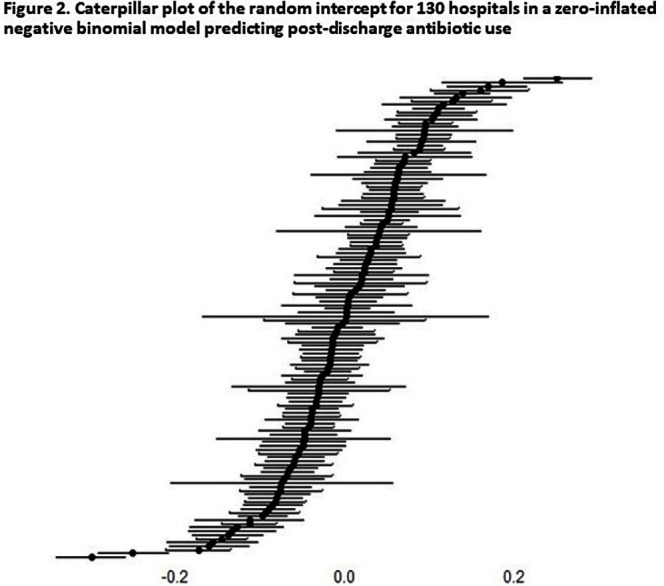# A novel risk-adjusted metric to compare hospitals on their antibiotic-prescribing at hospital discharge

**DOI:** 10.1017/ash.2024.123

**Published:** 2024-09-16

**Authors:** Daniel Livorsi, Jamie Merchant, Hyunkeun Cho, Matthew Goetz, Bruce Alexander, Brice Beck, Michi Goto

**Affiliations:** University of Iowa, Department of Internal Medicine; University of Iowa; UCLA/VA Greater Los Angeles Healthcare System; Iowa City VA Medical Center; Iowa City VA; University of Iowa Carver College of Medicine

## Abstract

**Background:** Approximately 40% of all antibiotics related to an acute-care hospital stay are prescribed at the time of hospital discharge. However, there is no metric to compare hospitals on their antibiotic-prescribing at this transition of care. In this study, we sought to build a risk-adjusted metric for comparing hospitals on their overall post-discharge antibiotic use. **Methods:** This was a retrospective study across all acute-care admissions within the Veterans Health Administration during 2018-2021. For patients discharged to home or self-care, data on antibiotics administered while inpatient and those prescribed at discharge were collected. To predict post-discharge antibiotic use (days of therapy, DOT), we built a zero-inflated negative binomial model with a random intercept for each VA medical center. Data were split into training and testing sets to measure model performance and absolute error. Covariates included patient demographics, medical specialty at discharge, comorbidities, discharge diagnoses of infection, and the length of inpatient antibiotic therapy. Outliers, defined as DOT ≥ 30, were excluded, and the predicted random intercept was used to determine hospital performance. To compare hospitals with a positive versus negative random intercept in our model (i.e. higher vs. lower than expected overall post-discharge use, respectively), we calculated mean total antibiotic duration (inpatient + post-discharge) for two uncomplicated infection types: community-acquired pneumonia (CAP) and skin and soft tissue infections (SSTI). **Results:** 1,804,400 patients were discharged to home or self-care across 130 hospitals. The mean age was 67.8 (SD 12.9), and 93.7% were male. Antibiotics were prescribed to 41.5% while hospitalized and 19.5% at discharge. The median number of post-discharge DOT among those prescribed post-discharge antibiotics was 7 (IQR 4-12). The predictive model detected post-discharge antibiotic use with fidelity, including accurate identification of any post-discharge antibiotic exposure (area under the precision-recall curve=0.97 ) and reliable prediction of the number of post-discharge DOT in those who were exposed (mean absolute error = 1.65; Figure 1). At negative versus positive random intercept hospitals (Figure 2), antibiotic duration for CAP and SSTIs was 7.3 versus 8.1 days (p < 0 .001) and 9.4 vs. 10.2 days (p < 0 .001), respectively. **Conclusion:** A model using electronically available data was able to accurately predict antibiotic use prescribed at hospital discharge. Hospitals with lower than expected overall post-discharge antibiotic use also prescribed shorter courses of antibiotic therapy for uncomplicated cases of CAP and SSTI, which may reflect more robust processes at these sites to reduce antibiotic overuse at discharge.

**Disclosure:** Michi Goto: Contracted Research – Merck

**Figure:**
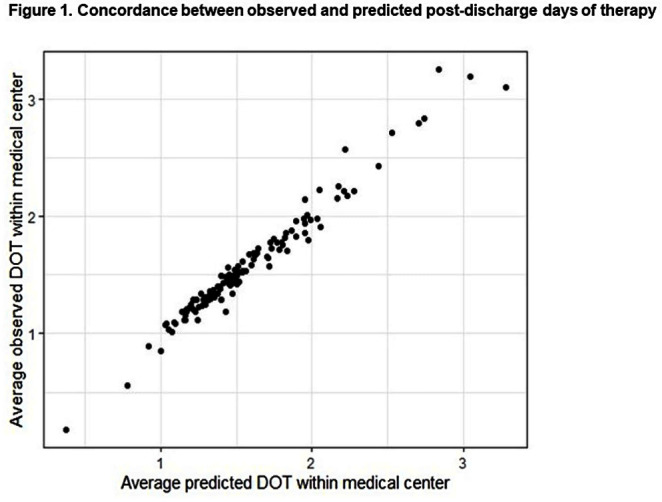

**Figure:**